# Absorbent Cotton

**Published:** 1883-08

**Authors:** 


### Absorbent Cotton.

Materials that will readily take up moisture are very important for the dentist's and surgeon's use. For this purpose, sponge, spunk, paper, lint, and cotton have been used. All of these materials are useful for certain cases. The sponge and lint are chiefly for the surgeon's purposes; spunk, paper, and cotton are more used by the dentist, and in minor surgical operations. The dentist should always have at hand all of these preparations, except, perhaps, the lint. Cotton or paper may be used by the dentist instead. The common unprepared cotton was formerly used. In this condition it is not a good absorbent. Some one, a few years ago, recommended its preparation by boiling in an alkaline solution. This greatly improves its absorbent properties. In this form it is now in the market, and for sale by all dealers in dental goods. It is prepared by several parties, but by none, perhaps, better than by the Dennison Manufacturing Company, who have agents in all the principal cities of the country. I have used this preparation for a year past, and regard as equal, if not superior, to any similar preparation.

This cotton has not only this property, but it is medicated as well for several purposes. It is a good vehicle for styptics. It may be prepared by saturating it in a strong solution of per sulphate of iron. Wring and dry, or use it moist. Carbolated cotton may be made by soaking it in a 33 per cent solution of carbolic acid and water. This should be used when in a moist condition. Salicylated cotton and borated cotton may be prepared in a similar manner, and they are desirable preparations as well.

